# Dissection of the *IDA* promoter identifies WRKY transcription factors as abscission regulators in Arabidopsis

**DOI:** 10.1093/jxb/erae014

**Published:** 2024-01-31

**Authors:** Sergio Galindo-Trigo, Anne-Maarit Bågman, Takashi Ishida, Shinichiro Sawa, Siobhán M Brady, Melinka A Butenko

**Affiliations:** Section for Genetics and Evolutionary Biology, Department of Biosciences, University of Oslo, Norway; Department of Plant Biology and Genome Center, University of California, Davis, CA, USA; International Research Organization for Advanced Science and Technology (IROAST), Kumamoto University, Kumamoto, Japan; Graduate School of Science and Technology, Kumamoto University, Kumamoto, Japan; Graduate School of Science and Technology, Kumamoto University, Kumamoto, Japan; Department of Plant Biology and Genome Center, University of California, Davis, CA, USA; Section for Genetics and Evolutionary Biology, Department of Biosciences, University of Oslo, Norway; University of Nottingham, UK

**Keywords:** Abscission, *cis*-elements, ERF, IDA, promoter, signaling, transcription, WRKY

## Abstract

Plants shed organs such as leaves, petals, or fruits through the process of abscission. Monitoring cues such as age, resource availability, and biotic and abiotic stresses allow plants to abscise organs in a timely manner. How these signals are integrated into the molecular pathways that drive abscission is largely unknown. The *INFLORESCENCE DEFICIENT IN ABSCISSION* (*IDA*) gene is one of the main drivers of floral organ abscission in Arabidopsis and is known to transcriptionally respond to most abscission-regulating cues. By interrogating the *IDA* promoter *in silico* and *in vitro*, we identified transcription factors that could potentially modulate *IDA* expression. We probed the importance of ERF- and WRKY-binding sites for *IDA* expression during floral organ abscission, with WRKYs being of special relevance to mediate *IDA* up-regulation in response to biotic stress in tissues destined for separation. We further characterized WRKY57 as a positive regulator of *IDA* and *IDA*-*like* gene expression in abscission zones. Our findings highlight the promise of promoter element-targeted approaches to modulate the responsiveness of the IDA signaling pathway to harness controlled abscission timing for improved crop productivity.

## Teaser

WRKY transcription factor-binding sites contribute to the transcriptional regulation of *IDA* . WRKY57 modulates abscission via redundant IDA/IDA-like peptides.

## Introduction

Abscission is a developmentally programmed process of cell separation. Indeterminate growth and a modular developmental plan allow plants to shed organs that are no longer needed. Abscission can take place in leaf petioles, floral organs, flower pedicels, fruits, or seeds, to name a few. The ubiquitous presence of abscission across plant organs and developmental phases provides these sessile organisms with flexibility to prioritize resource allocation and a very effective strategy to minimize disease spread. On the other hand, untimely or uncontrolled abscission has profound negative consequences for agriculture. Indeed, as humankind has domesticated plants, seed abscission has been selected against in crops, hindering a process that naturally evolved to aid seed dispersal for the benefit of more efficient and plentiful harvests (Pickersgill, 2007). Controlled abscission is still actively sought after in breeding programs. Understanding the complex set of cues that influence abscission occurrence or its timing is thus highly relevant.

There is a broad spectrum of cues that influence abscission. The phytohormones auxin and ethylene have antagonistic effects on abscission (Addicott *et al.*, 1955; Abeles and Rubinstein, 1964; Louie and Addicott, 1970; Beyer and Morgan, 1971; Meir *et al.*, 2006, 2010, 2015; Basu *et al.*, 2013). The generally accepted model suggests that the competence to abscise is blocked by auxin efflux from the organ into the abscission zone (AZ). Ethylene is a positive effector of abscission, and ethylene sensitivity acquisition is a milestone for abscission induction (Abeles and Rubinstein, 1964; Burg, 1968; Reid, 1985; Brown, 1997; Dal Cin *et al.*, 2005; Merelo *et al.*, 2017; Botton and Ruperti, 2019). Carbohydrate availability is a well-known factor regulating abscission induction (Sawicki *et al.*, 2015). Carbohydrate starvation triggers abscission, as documented in multiple plant species after shading or defoliation (Addicott *et al.*, 1955; Aloni *et al.*, 1997; Peng *et al.*, 2013). Exogenous cues such as water availability and pathogens also regulate abscission induction (Patharkar and Walker, 2016; Reichardt *et al.*, 2020). Plants sense infections in leaves and trigger abscission to diminish the spread of the disease (Ketring and Melouk, 1982; Ben-David *et al.*, 1986; Glick *et al.*, 2009; Scalschi *et al.*, 2014). Other phytohormones (cytokinin, salicylic acid, and jasmonic acid), developmental cues (senescence, and fruit and seed development), as well as exogenous cues (light and temperature) are known to influence abscission (for a review, see Ma *et al.*, 2021). Understanding how the molecular pathways that execute abscission integrate these complex signals would greatly inform abscission-related breeding programs.

The abscission of floral organs in *Arabidopsis thaliana* (Arabidopsis) is the best characterized abscission model. AZs develop at the base of each floral organ (sepals, petals, and stamens) as two adjacent but distinct cell layers: the residuum and secession layers. Secession cells are located proximal to the abscising organ and form a lignified structure called the lignin brace. The lignin brace is thought to focus cell wall-degrading enzyme activity and provide local rigidity to facilitate shedding (Lee *et al.*, 2018). Residuum cells make up the cell layer that remains on the flower receptacle after abscission occurs, differentiating into cuticle-bearing epidermal-like cells. Several transcription factors (TFs) are necessary for AZs to differentiate during flower development, including BLADE ON PETIOLE 1 (BOP1) and BOP2, ARABIDOPSIS THALIANA HOMEOBOX GENE 1 (ATH1), KNOTTED-LIKE FROM ARABIDOPSIS THALIANA 2 (KNAT2), and KNAT6 (McKim *et al.*, 2008; Crick *et al.*, 2022). When AZs have developed and floral organs are no longer required, AZs secrete the peptide INFLORESCENCE DEFICIENT IN ABSCISSION (IDA) to trigger cell separation (Butenko *et al.*, 2003). IDA is perceived in the AZ cells by the leucine-rich repeat (LRR) receptor kinases (RKs) HAESA (HAE) and HAESA-LIKE 2 (HSL2) and their co-receptors, members of the family of somatic embryogenesis receptor kinases (SERKs; Jinn *et al.*, 2000; Cho *et al.*, 2008; Stenvik *et al.*, 2008; Meng *et al.*, 2016; Santiago *et al.*, 2016). When the HAE–SERK/HSL2–SERK receptor complexes activate, they trigger an intracellular signaling cascade of MITOGEN-ACTIVATED PROTEIN KINASES (MAPKs; Cho *et al.*, 2008; Zhu *et al.*, 2019). The MAPK cascade inhibits negative transcriptional regulators of abscission, KNAT1 and AGAMOUS-LIKE 15, thereby allowing the progression of abscission (Wang *et al.*, 2006; Shi *et al.*, 2011; Butenko *et al.*, 2012; Patharkar and Walker, 2015). Other regulators influence abscission indirectly by modulating the IDA-induced signaling pathway (Liljegren *et al.*, 2009; Leslie *et al.*, 2010; Burr *et al.*, 2011; Liu *et al.*, 2013; Gubert and Liljegren, 2014; Baer *et al.*, 2016; Taylor *et al.*, 2019).

The signaling cascade induced by IDA and HAE/HSL2 in AZs is a requisite for floral organ abscission. Double mutants *hae hsl2* retain all floral organs across floral positions in the inflorescence (Cho *et al.*, 2008; Stenvik *et al.*, 2008). Meanwhile, *ida* knockouts display a weaker abscission phenotype, with floral organs being loosely attached in floral positions in which siliques are elongating (Stenvik *et al.*, 2008; Alling and Galindo-Trigo, 2023). The weaker abscission phenotype in *ida* mutants is probably due to functional redundancy with related IDA-like (IDL) peptides in AZs (Stenvik *et al.*, 2008; Vie *et al.*, 2015). A recent review of the literature has proposed that the IDA pathway could be responsible for the very last steps of separation, mediating an increase in turgidity and cell expansion, while abscission activation and initiation of cell wall degradation would be mostly dependent on ethylene signaling (Meir *et al.*, 2019). In this scenario, IDA would be one of several responses that ethylene activates in AZs to orchestrate the separation of floral organs. AZ promoter activity of *IDA* was indeed found to depend on ethylene signaling (Butenko *et al.*, 2006). Wounding was also shown to induce early activation of the *IDA* promoter in AZs (Butenko *et al.*, 2006). In cauline AZs, drought stress induces *IDA* transcription (Patharkar and Walker, 2016). In the lateral root emergence zone of seedlings, *IDA* promoter activity is enhanced in response to the microbe-associated molecular patterns (MAMPs) flagellin (flg22) and chitin, as well as to salt and mannitol (Lalun *et al.*, 2023). These instances suggest that a sizeable set of environmental factors known to influence abscission are integrated in the transcriptional regulation of *IDA*.

In this study, we investigate the genetic and molecular determinants of *IDA* transcriptional regulation. An *in silico* dissection of the *IDA* promoter and a screen against an Arabidopsis TF collection highlight the diversity of cues and effectors that can modulate *IDA* expression. We investigated the relevance of an ethylene response factor- (ERF) binding site for *IDA* promoter activity. Further, we demonstrate that several DNA-binding sites of WRKY TFs are required for full transcriptional competence of *IDA* and to mediate its MAMP-dependent transcriptional up-regulation, respectively. We also show that WRKY57 can modulate floral organ abscission in an IDA/IDL- and HAE/HSL2-dependent manner.

## Materials and methods

### Plant material, growth conditions, and treatments

All Arabidopsis lines were in the Columbia (Col-0) genetic background, except for the *ida-1* mutant (C24; Butenko *et al.*, 2003). The previously published mutant and transgenic lines used in this study were: *ida-2* (Cho *et al.*, 2008), *idaCR* (Alling and Galindo-Trigo, 2023), *idl1CR* (Shi *et al.*, 2018), *p35S::IDA* (Stenvik *et al.*, 2006), *hae hsl2* (Stenvik *et al.*, 2008), *wrky57* (Jiang *et al.*, 2012), *wrky60-1* (Xu *et al.*, 2006), and *wrky48* (Jiang *et al.*, 2012). Genotyping primers are listed in Supplementary Table S1.

Arabidopsis seeds were routinely vapor-sterilized with chlorine gas, sown on Murashige and Skoog (MS) medium plates with 0.7% sucrose, stratified for 3 d, and germinated in growth chambers for a week before transfer to regular sowing soil. Subsequently, plants grew in climate rooms until seed setting and senescence. Environmental growth conditions in the growth chamber and climate rooms were similar: a photoperiod of 16 h day/8 h night, light intensity of 130–150 μmol m^–2^ s^–1^, temperature of 22 °C, and 60% humidity. Transgenic plants were selected in plates supplemented with Basta or hygromycin-B as required. Microscopy of roots was carried out with vertically grown seedlings in 0.5× MS plates with 0.7% sucrose.

In the case of the flg22-treated mature rosette leaves, seeds were directly germinated on peat pellets (Jiffy 7) with a short-day photoperiod (10 h day/14 h night) to allow the development of multiple fully expanded rosette leaves of comparable size per plant prior to bolting. Two plants per pellet were allowed to progress past the seedling stage. At week 6 and prior to bolting, the most expanded rosette leaf of each plant was syringe infiltrated with mock solution (water) or flg22 solution (500 nM flg22 in water). Twenty hours later, leaves were detached and individually processed to detect β-glucuronidase (GUS) activity. Leaves were assigned to different qualitative categories with values ranging from 0 (undetectable GUS staining) to 3 (strong GUS signal in the vasculature and neighboring leaf tissues). To quantify the effect of flg22 on root meristems, seedlings were germinated in liquid MS as in Luna *et al.* (2011). Liquid medium was refreshed after 1 week of growth, and flg22 treatments were applied 1 d later. Mock treatments (water) or flg22 treatments (1 µM flg22 in water) were applied in the evening of the eighth day, and seedlings were processed for microscopy the following day after ~20 h of treatment. Quantification of *IDA* induction in root meristems was conducted by producing maximum intensity projections of the H2B-TdTomato channel images, and counting the total number of fluorescent nuclei in the meristematic region using the StarDist plugin in ImageJ with pre-determined settings (Schmidt *et al.*, 2018). To assay the responsiveness of AZs to flg22, plants were grown to maturity in standard conditions. Developing siliques in positions 6–7 that had already shed all floral organs were selected and separated from the plant by the pedicel. The siliques were immediately submerged in the mock (water with 0.02% Silwet L-77) or flg22 treatments (10 µM flg22, 0.02% Silwet L-77 in water) for 15 min. Subsequently, the siliques were transferred to the overnight incubation solutions for mock (water) or flg22 treatments (10 nM flg22 in water). The initial short treatment with detergent allows for the explants to become less hydrophobic and an even elicitation. Incubation overnight in the solutions without detergent helps avoid toxicity. Cauline leaf elicitation with flg22 was conducted on 5-week-old plants grown under standard conditions. The first two cauline leaves of each plant were syringe-infiltrated with mock solution (water) or flg22 solution (1 µM flg22 in water). The entire surface of the leaf was infiltrated, including the boundary between the leaf and stem. The treatment was allowed to proceed for 20 h. The cauline leaf–stem boundary was manually dissected with a razor blade and imaged with confocal microscopy immediately after.

*Nicotiana benthamiana* plants were grown in growth chambers with a long-day photoperiod, diurnal temperature of 22 °C, and night temperature of 19 °C, 150–180 μmol m^–2^ s^–1^ of light, and 60% relative humidity.

### Generation of genetic constructs and transgenic lines

Arabidopsis plants were transformed with *Agrobacterium* C58 following the floral dip method (Clough and Bent, 1998).

Most genetic constructs used in this study were generated with Invitrogen Gateway recombination cloning. Promoter sequences were cloned into the cloning vector pENTR5' (TOPO-TA cloning; Invitrogen). The *IDA* promoter was cloned from C24 gDNA as the 1417 bp between the *IDA* translation initiation site (TIS) and the upstream gene *AT1G68780*. *IDA* promoter sequences in C24 and Col-0 accessions are identical, with the exception of the length of a dinucleotide repeat located 144 bp from the TIS that is extended by 12 bp (CACACACACAAG) in the C24 genome. In all other cloning instances, the Col-0 accession was used as template.

The *ERF*(–) and *WRKY*(–) versions of the *IDA* promoter were generated by site-directed mutagenesis (Zheng *et al.*, 2004). The *ERF*(–) version of the *IDA* promoter carries a 33 bp deletion centered on the –305 bp ERF TF-binding site (TFBS) that removes the predicted binding site of 66 ERFs (Supplementary Table S2). Aside from the –305 bp ERF TFBS, this deletion also disrupts the predicted binding site of four TFs not detected in the yeast one-hybrid (Y1H) assay and not expressed/weakly expressed in AZ cells according to Cai and Lashbrook (2008) and Lee *et al.* (2018) in AZ transcriptomic databases [*AT2G31220* (not expressed), *AT2G15740* (not expressed), *AT5G22990* (not expressed), *AT5G28300* (weakly expressed)], as well as a WRKY TFBSs targeted in the *WRKY*(–) promoter. Importantly, this particular WRKY TFBS also overlaps with the TFBS of the weakly expressed *AT5G28300*. Disrupting the WRKY TFBS located at –300 bp (and therefore also the predicted TFBS of *AT5G28300*) did not yield any noticeable decrease in *IDA* expression, suggesting that the functionally important *cis*-element missing in *ERF*(–) is the –305 bp ERF TFBS.

To generate the *WRKY*(–) *IDA* promoter, WRKY TFBSs were disrupted by exchanging the five nucleotides of each predicted WRKY recognition motif for TTTTT, therefore minimizing the disruption of neighboring TFBSs. Five consecutive rounds of site-directed mutagenesis were necessary to generate the *WRKY*(–) *IDA* promoter. The sequences of the *ERF*(–) and *WRKY*(–) promoters can be found in Supplementary Table S3.

The *IDL1* promoter comprises 1557 bp between its TIS and the upstream gene *AT3G25660*. The *IDL2* promoter comprises 2084 bp upstream of its TIS. The *IDL3* promoter comprises 1974 bp upstream of its TIS. The *WRKY57* promoter covers 2071 bp upstream of its TIS. The *WRKY60* promoter comprises 1512 bp between its TIS and the upstream gene *AT2G24990*. The *HAESA* promoter contains 1729 bp upstream of the 37th base pair from the TIS. Coding sequences to be expressed *in planta* were cloned into pDONR221 or pDONR-Zeo in BP Clonase II reactions (Invitrogen). Intronless coding sequences from *IDA*, *WRKY57*, and *WRKY60* were PCR amplified from floral tissue cDNA [see the section on quantitative reverse–transcription PCR (RT–qPCR) for methods regarding RNA extraction and cDNA synthesis]. The WRKY57srdx entry vector was obtained by amplifying the *WRKY57* coding sequence from floral cDNA using a modified reverse primer containing the coding sequence of the repressor motif SRDX (CTCGATCTGGATCTAGAACTCCGTTTGGGTTTCGCT) in-frame with the C-terminus of *WRKY57*. These entry vectors were recombined into destination vectors from the Nakagawa lab (Nakagawa *et al.*, 2007, 2008; Tanaka *et al.*, 2013) by means of LR recombination reactions using either LR II Clonase or LR II Clonase Plus (Invitrogen). The destination vector used to generate *GFP-GUS* promoter reporter lines was R4L1pGWB632. Luciferase promoter reporters were generated with the destination vector R4L1pGWB635. Green fluorescent protein (GFP)-tagged translational fusion reporters were cloned into R4pGWB504. Non-C-terminally tagged *promoter*:*cds* constructs were cloned into R4pGWB501. Effector constructs to overexpress TFs or negative controls in luciferase assays were cloned into pGWB518.

To generate nuclear fluorescent promoter reporters *H2B-TdTomato* and *Venus-H2B*, a pDONR221 entry clone containing the coding sequence of the fusion proteins H2B-TdTomato or Venus-H2B were first generated. The *H2B-TdTomato* sequence was PCR amplified from pAH21-H2B-TdTomato, while the *Venus-H2B* sequence was amplified from pAB146 (kindly provided by Simon Rüdiger). These entry clones were subsequently recombined with the corresponding promoter and/or destination vectors.

Col-0 wild-type plants were gene edited to produce the *idl2CR* and *idl3CR* lines with the plasmid system of Fauser *et al.* (2014) comprising pDe-Cas9 and pEn-Chimera vectors. Guide RNA protospacers targeting *IDL2* in its 69th codon (*Bmg*BI restriction site) and *IDL3* in its 37th codon (*Xmn*I restriction site) were designed. Transformants were screened by the cleaved amplified polymorphic sequence (CAPS) method, and mutant alleles were confirmed by Sanger sequencing. A +1 bp insertion causes a frameshift immediately prior to the IDL2 peptide in *idl2CR* plants, whereas in *idl3CR* plants a –1 bp deletion causes a frameshift in the variable region of the IDL3 protein. The Cas9 T-DNA cassette was segregated away from the plant genetic background, and homozygous plants for the *idl2CR* or *idl3CR* mutations were confirmed by CAPS and sequencing.

Constructs used in the Y1H assay were generated by recombining in Gateway LR reactions the *IDA* promoter entry vector with the two yeast reporter plasmids—pMW2 and pMW3 (Deplancke *et al.*, 2006)—containing, respectively, HIS3 or LacZ reporter genes.

Primers used to generate these constructs can be found in Supplementary Table S1.

### Detection and visualization of expression reporters

Promoter activity in GUS (Jefferson *et al.*, 1987) reporter lines was detected as follows: explants/seedlings were incubated in ice-cold 90% acetone for 20 min, washed for 20 min in staining buffer without X-Gluc (50 mM NaPO_4_ pH 7.4, 2 mM potassium ferro-cyanide, 2 mM potassium ferri-cyanide, 0.1% Triton X-100), and stained in staining buffer with 2 mM X-Gluc at 37 °C for 3 h or overnight. The chlorophyll was then cleared from the tissues with washes in 75% ethanol for 1–3 d and imaged on a stereomicroscope or widefield microscope.

Fluorescent reporter detection was conducted on live tissue. Explants were mounted in water and immediately imaged in a Zeiss LSM880 confocal microscope. To visualize GFP-tagged translational fusions as well as the GFP–GUS dual reporter or Venus-H2B, fluorophores were excited with a laser wavelength of 488 nm. The nuclear transcriptional reporter H2B-TdTomato was excited with a wavelength of 561 nm. To co-localize nuclear reporters (GFP or H2B-TdTomato) with DAPI, live explants were incubated in DAPI staining solution (0.2 mg l^–1^ in water) for 30 min and subsequently imaged with an excitation wavelength of 405 nm. Chlorophyll autofluorescence was excited with a laser wavelength of 633 nm to allow easier identification of AZ or cauline leaf regions.

### Abscission zone and cell size estimations

AZs of 6-week-old plants grown in standard conditions were imaged in a stereomicroscope. The transversal area occupied by the receptacle was manually selected in ImageJ (Schneider *et al.*, 2012), and then measured. To estimate the cell size in AZs, the AZs were stained with propidium iodide (10 mg l^–1^ in water) and imaged in a Zeiss LSM880 confocal microscope with laser excitation at 561 nm. Concomitantly, the GFP-tagged proteins were imaged with an excitation wavelength of 488 nm. For each AZ, the longest possible diameter of five consecutive cells from the sepal AZ was measured manually with ImageJ and then averaged to produce the mean estimated cell size of an AZ. Transversal AZ area was measured in position 12 siliques. Cell size was measured in position 8. These positions displayed the greatest contrast in size between wild-type plants and genotypes in which AZs enlarged, while technically allowing the imaging to be conducted reliably.

### Gene expression estimations by RT–qPCR

Floral receptacles were manually dissected from at least 25 flowers (positions 5/6) collected from 3–4 plants to yield each biological replicate. Excised receptacles were immediately transferred to tubes pre-chilled in liquid nitrogen, and flash-frozen in liquid nitrogen. RNA was extracted using either the Spectrum Plant Total RNA Kit (Sigma) or RNeasy Plant Mini Kit (Qiagen) following the manufacturer’s instructions and including an on-column DNase I digestion step (Sigma). First-strand cDNA was synthesized with Superscript III or Superscript IV Reverse Transcriptase (Invitrogen), RNA was digested with RNase H, and selected loci were amplified and quantified with FastStart Essential DNA Green Master (Roche) in a LightCycler96 instrument (Roche). Gene expression was estimated with the 2^–ΔCt^ method using *ACTIN2* as a reference gene. Experiments comprised three biological replicas per genotype and two technical replicas per RT–qPCR. Primers used in RT–qPCRs can be found in Supplementary Table S1.

### Luciferase promoter transactivation assays

Transactivation assays in transiently transformed *N. benthamiana* plants were carried out according to Lasierra and Prat (2018). Briefly, fully expanded leaves from 4-week-old plants were syringe-infiltrated with *Agrobacterium* solutions carrying the reporter and effector plasmids at 0.02 OD_600_ each. Measurements were conducted 3 d after infiltration on a 96-well OptiPlate (PerkinElmer) by floating 4 mm diameter leaf discs (abaxial side up) on 200 µl of luciferin solution (1× MS salts, 0.5% MES, pH 5.8, 12 µM d-luciferin). Light emission was measured in a Wallac 1420 VICTOR2 microplate reader (PerkinElmer) recording the light emitted for 10 s, with a 10 min delay between each repeat. The third plate repeat after ~35 min typically recorded the strongest signal and was used to plot and analyze differences between constructs. Twelve leaf discs per construct combination coming from two different plants per construct combination were assayed per experiment.

Assays with the *IDA* promoter luciferase constructs were first attempted in *N. benthamiana* leaves, but strong autoactivation of the *IDA* promoter in this system impeded reliable quantifications. An Arabidopsis mesophyll protoplast transient transfection system was used instead. We used fully expanded rosette leaves from pre-bolting, 4-week-old Col-0 plants grown in our standard conditions to extract the protoplasts following the tape-sandwich method (Wu *et al.*, 2009; Hansen and van Ooijen, 2016). Transfections were carried out with 50 µl of protoplasts at 400 000 protoplasts ml^–1^ and 6 µg of plasmid DNA, and purified with the PureLink HiPure Plasmid Midiprep Kit (Thermofisher). After transfections, protoplasts were allowed to recover in W5 solution overnight. The protoplasts were gently pelleted and resuspended in 100 µl of W5 solution with 1 mM d-luciferin and immediately transferred to 96-well plates. Light emission was recorded for 5 s, with an 8 min delay between each repeat. Four independent protoplast transfections per construct combination were analyzed per experiment.

### *In silico* analyses of promoter sequences

The promoter sequence of *IDA* was scouted for the presence of TFBSs using the Binding Site Prediction tool from the Gao lab’s PlantRegMap site (http://plantregmap.gao-lab.org/; Supplementary Table S2; Tian *et al.*, 2019). Gene Ontology (GO) term enrichment analysis was carried out using the GO Term Enrichment tool in PlantRegMap. The input was the list of TFs identified as *IDA* promoter interactors in either HIS3 or LacZ assays, and the reference set of genes was the total list of TFs from *A. thaliana*—downloaded from the PlantRegMap site. This tool calculates statistically significant enrichment with topGO and Fisher’s exact tests, with threshold *P*-value ≤0.01. Additional TFBS searches were conducted with the PlantPAN 3.0 web tool (http://plantpan.itps.ncku.edu.tw/plantpan4; Chow *et al.*, 2019).

### Yeast one-hybrid screen

The *IDA* promoter was recombined in the pMW2 and pMW3 plasmids, respectively, to drive expression of the HIS3 and LACZ reporters (Gaudinier *et al.*, 2017). These reporter plasmids were transformed into the yeast YM4271 strain and the yeast colonies were screened for autoactivation and the construct presence via PCR genotyping. The Enhanced Yeast One-Hybrid screening of the *IDA* promoter against a complete collection of 2000 Arabidopsis TFs was done as described previously (Gaudinier *et al.*, 2011, 2017; Pruneda-Paz *et al.*, 2014). The positive interactions were recorded for LacZ and HIS3 activity. The Y1H screening was carried out by the Yeast One Hybrid Services Core at the UC Davis Genome Center, at the University of California, Davis (https://genomecenter.ucdavis.edu/yeast-one-hybrid-services). Y1H screening results are listed in Supplementary Table S4.

### Floral organ retention quantification

To phenotype and quantify abscission, plants were grown in individual pots, and plants from different genotypes were shuffled in their positions across the growth tables to minimize positional effects. Plants were grown undisturbed and untouched to minimize uneven shedding of floral organs prior to phenotyping. Phenotyping was generally conducted at week 6 when inflorescences had produced between 20 and 25 flowers post-anthesis. Floral organ abscission was quantified by counting the floral organs attached to the flowers in the main inflorescence according to Alling and Galindo-Trigo (2023). The main inflorescence stem was shaken four times, and the number of floral organs that remained attached to each floral position (P1–P20) was visually inspected.

## Results

### Dissection of promoter *cis*-elements suggests that the *IDA* gene is subject to intricate transcriptional regulation

One of the main genomic features that dictate the expression profile of a gene is the presence of *cis*-regulatory sequences to which TFs specifically bind (TFBSs). We used the web-based PlantTFDB 4.0 database to determine the presence of conserved TFBSs in the promoter of *IDA* to investigate the determinants of its transcriptional regulation (Jin *et al.*, 2017). We detected TFBSs for all the major TF families in plants, including ERF and WRKY, among others (Supplementary Fig. S1; Supplementary Table S2). The majority of the TF families detected presented one or more TFBSs within 500 nucleotides of the TIS of *IDA*, the portion of promoters shown to withstand the most stringent evolutionary constraints in a panel of Arabidopsis accessions (Korkuc *et al.*, 2014). This suggests that the transcription of *IDA* may be effectively controlled by an extensive array of TFs, allowing for its spatially and temporally restricted, yet highly environmentally responsive, expression pattern (Butenko *et al.*, 2003, 2006; Vie *et al.*, 2015; Patharkar and Walker, 2016; Lalun *et al.*, 2023).

**Fig. 1.**
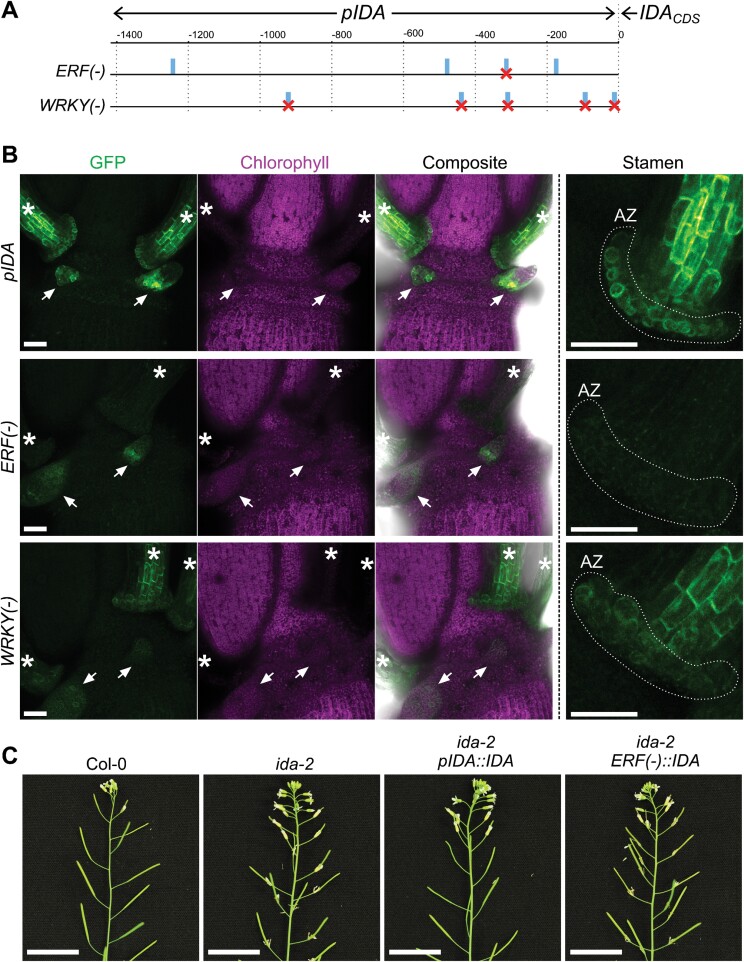
The *IDA* promoter activity depends on the presence of several TFBSs. (A) Schematic diagram of the *IDA* promoter sequence and the two mutant versions: *ERF*(–) and *WRKY*(–). Numbers in the upper scale represent the distances in number of nucleotides from the translation initiation site of the *IDA* gene. Blue rectangles indicate the predicted TFBSs for ERF or WRKY TFs along the *IDA* promoter. Crosses mark the specific promoter sites that were disrupted by site-directed mutagenesis in either version of the *IDA* promoter [see also the Materials and methods for additional details on the nature of the *ERF*(–) deletion and additional *cis*-elements disrupted]. (B) Representative confocal micrographs of plants expressing GFP–GUS. Each row corresponds to a promoter reporter line, indicated on the left. GFP is shown in green; chlorophyll autofluorescence is shown in magenta. The rightmost column shows detailed images of the stamen proximal region and AZ. White dotted lines highlight the AZ region from which quantitative measurements in Supplementary Fig. S2B were taken. White arrows point towards nectaries. White stars highlight stamens. Scale bars at both magnifications represent 50 µm. (C) Floral organ abscission phenotypes of *ida-2* mutants genetically complemented with the *IDA* gene under its wild-type promoter (*pIDA*) or the *ERF*(–) mutant version. Floral organs remain attached to most developing siliques in the *ERF*(–) lines, indicating lack of complementation of the *ida-2* abscission defect. Twelve out of 14 *pIDA* lines fully reverted the *ida-2* phenotype. Zero out of 20 *ERF*(–) lines fully reverted the *ida-2* phenotype. See also Supplementary Fig. S4. Scale bars are 2 cm.

Given the extensive list of potentially important TFBSs (Supplementary Table S2), we decided to conduct proof-of-concept experiments on ERF- and WRKY-binding sites to functionally demonstrate the physiological relevance of TFBSs for *IDA* expression. Members of the ERF and WRKY TFs regulate physiological processes adjacent to *IDA* signaling and abscission such as ethylene signaling, drought responses, MAMP-induced responses, or senescence (Lorenzo *et al.*, 2003; Zhou *et al.*, 2011; Jiang *et al.*, 2012; Chang *et al.*, 2013; Cheng *et al.*, 2013; Lyons *et al.*, 2013; He *et al.*, 2016; Jiang and Yu, 2016; Lal *et al.*, 2018). We used site-directed mutagenesis to disrupt selected TFBSs for ERFs and WRKYs in the *IDA* promoter, and observed the effect of the mutations on the expression of the promoter with a GUS–GFP dual reporter in stably transformed Arabidopsis plants (Tanaka *et al.*, 2013). Disruption of a single ERF TFBS predicted to convey signals of up to 66 ERF TFs was sufficient to reduce the activity of the *IDA* promoter to barely detectable levels in AZs and floral organs [*ERF*(–); Fig. 1A, B; Supplementary Fig. S2; see also additional remarks in the Materials and methods]. The *ERF*(–) *IDA* promoter was also inactive during lateral root emergence (Supplementary Fig. S3A). We then tested its capacity to genetically complement the abscission phenotype of *ida* knockouts (Butenko *et al.*, 2003; Cho *et al.*, 2008). The *ERF*(–) promoter was unable to revert the abscission phenotype of *ida*, indicating that its weak activity in nectaries does not induce floral organ separation (Fig. 1C; Supplementary Fig. S4). These results suggest that ERF-mediated signaling could be crucial for *IDA* expression and required for abscission to take place. On the other hand, disrupting the three WRKY TFBSs predicted with our initial search did not yield noticeable changes to the promoter activity (Supplementary Fig. S1). Additional WRKY TFBSs were found in the *IDA* promoter by alternative bioinformatic tools, and a quantifiable decrease in the promoter activity in AZs was observed when five binding sites were mutated [*WRKY*(–); Fig. 1A, B; Supplementary Figs S2, S5]; (Chow *et al.,* 2019). Despite its decreased activity in AZ cells, the *WRKY*(–) *IDA* promoter was still active in floral organs and during lateral root emergence, suggesting that WRKY TFs have an important but not essential role in the developmentally induced expression of *IDA* (Supplementary Fig. 3B).

### WRKY-binding sites are necessary to activate the *IDA* promoter in response to the immunity elicitor flg22

**Fig. 2.**
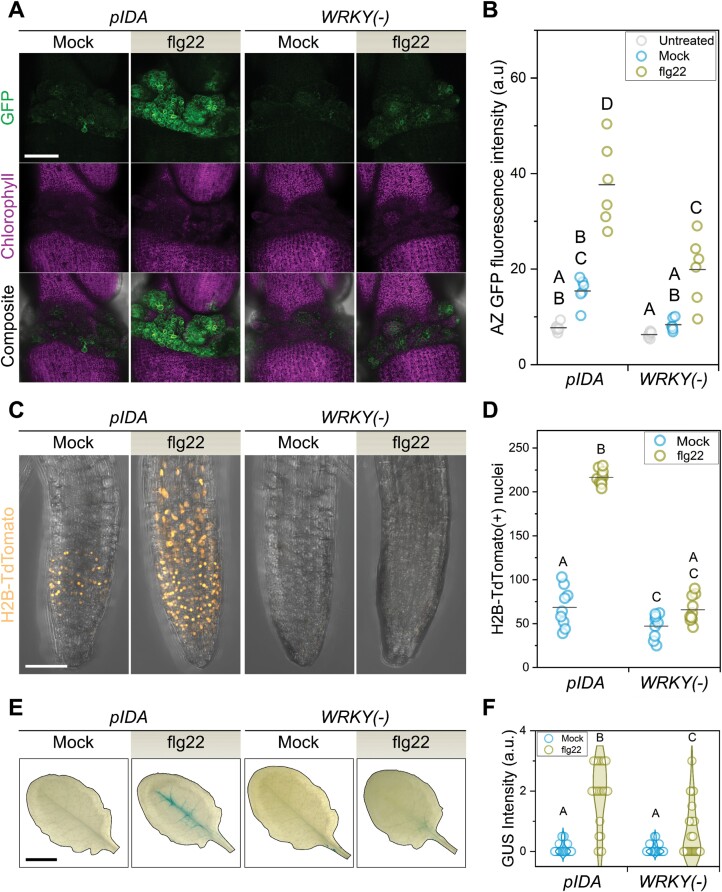
*IDA* responsiveness to flg22 depends on the presence of WRKY-promoter binding sites. (A) Residuum AZ cells induce *IDA* in response to flg22 in a WRKY TFBS-dependent manner. Reporter lines express GFP–GUS under the control of the promoters indicated on the top. Scale bar=120 µm. Micrographs shown are maximum intensity projections of the GFP, chlorophyll autofluorescence, or composite channels. Images are representative of an experiment with six plants per line, and one flower in positions 6 or 7 per plant per treatment. The experiment was repeated twice using different transgenic lines for each reporter construct with similar results. (B) Quantification of the GFP fluorescence intensity in the entire AZ area of the maximum intensity projections shown in (A). Each data point corresponds to the average GFP intensity in the AZ region of one flower. (C) The root meristematic region responds to flg22 treatment by up-regulating *IDA* in a WRKY TFBS-dependent manner. Reporter lines express H2B-TdTomato under the control of the promoters indicated on the top. Images are maximum intensity projections of composite confocal micrographs. Scale bar=30 µm. Images are representative of an experiment with 10 seedlings per line and treatment. The experiment was repeated three times with three independent lines per reporter construct, and similar results were obtained. (D) Quantification of nuclei with H2B-TdTomato expression from maximum intensity projections of the root meristematic region. Each data point corresponds to the nuclei quantified in each individual seedling assayed. (E) Representative images of the histochemical detection of GUS activity in mature rosette leaves in plants expressing GFP–GUS under the promoters displayed on the top. Scale bar=0.5 cm. (F) Quantification of the GUS activity detected in mature leaves like those in (E). Data shown here contain the dataset from three independent transgenic lines per reporter construct, totaling between 24 and 32 plants per construct and treatment combination. Leaf GUS staining intensity was qualitatively assessed and assigned to categories (see the Materials and methods). Letters in (B), (D), and (F) represent categories of statistically significant differences according to two-way ANOVA and post-hoc pairwise comparisons with Bonferroni tests (*P<*0.05).

When plants are compromised by infections, organs that would otherwise be retained are abscised to protect the plant (Lahey *et al.*, 2004; Scalschi *et al.*, 2014; Patharkar *et al.*, 2017). It is well established that bacterial and fungal elicitors induce *IDA* expression; however, the molecular mechanism driving this transcriptional up-regulation is not known (Vie *et al.*, 2015; Lalun *et al.*, 2023). Several WRKY TFs are elicited by MAMPs such as flg22, are capable of inducing transcription of defense-related genes, and their overexpression enhances disease resistance (Asai *et al.*, 2002; Navarro *et al.*, 2004; Zipfel *et al.*, 2004; Birkenbihl *et al.*, 2016). We thus hypothesized that WRKY TFBSs in the *IDA* promoter could influence its responsiveness to pathogenic cues. Indeed, *WRKY*(–) reporter lines were significantly less responsive than lines with the wild-type *IDA* promoter to treatments with flg22 in multiple tissues (Fig. 2). Wild-type *IDA* promoter was strongly activated by flg22 in AZ cells, whereas its *WRKY*(–) counterpart only showed a weak induction (Fig. 2A, B). In seedlings, flg22 treatment also induced the *IDA* promoter in the meristematic zone of the main root, while the *WRKY*(–) *IDA* promoter was not induced (Fig. 2C, D). The same result was observed when fully expanded rosette leaves were infiltrated with flg22, as the observed induction of the *IDA* promoter along the leaf midrib was reduced in the *WRKY*(–) reporter lines (Fig. 2E, F). The receptors of IDA, HAE and HSL2, are involved in pathogen-induced cauline leaf abscission in Arabidopsis (Patharkar *et al.*, 2017). We therefore looked for up-regulation of the *IDA* promoter in cauline AZs after infiltrating cauline leaves with flg22. We did not detect *IDA* promoter activity in this tissue under our conditions, and thus we hypothesize that other *IDL* genes that are responsive to flg22, such as *IDL6* and/or *IDL7*, could be responsible for bacteria-induced cauline leaf shedding (Supplementary Fig. S6). Our results support a role for WRKY TFs in mediating *IDA* expression upon biotic stress in floral AZs, roots, and rosette leaves. We propose the *IDA* promoter to be a direct target of immunity-activated WRKYs (Asai *et al.*, 2002; Lal *et al.*, 2018).

### WRKY57 is a potential regulator of *IDA* transcription

**Fig. 3.**
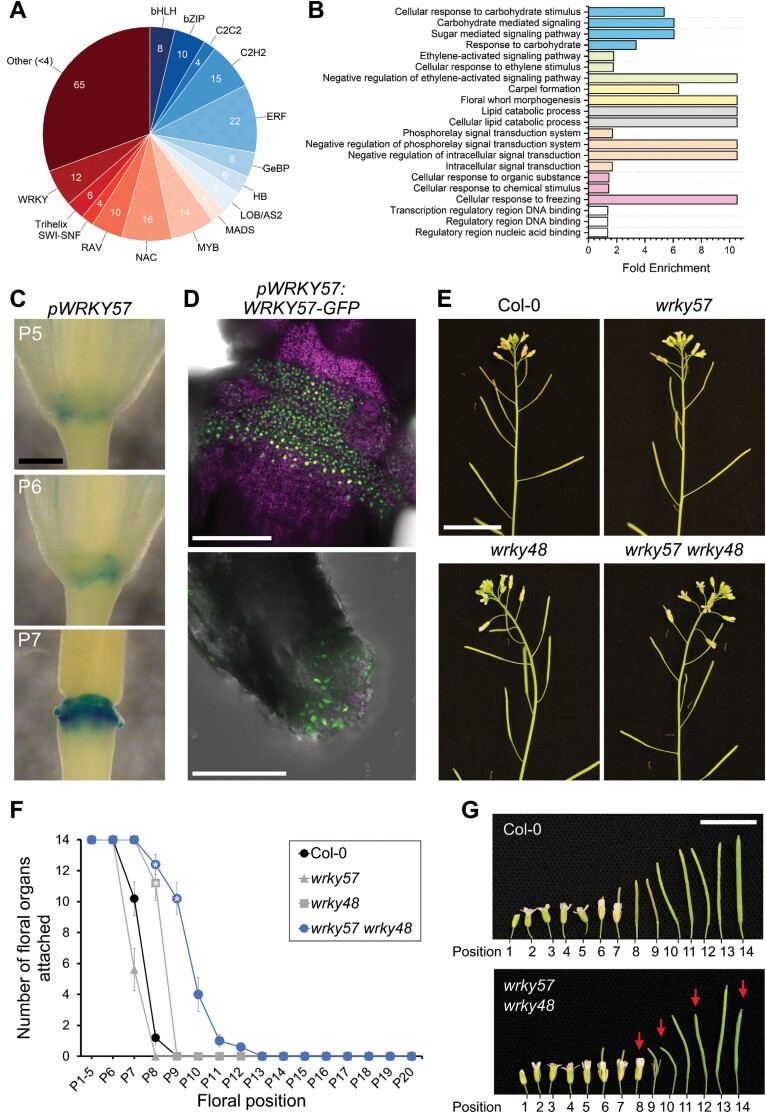
A screening against a collection of Arabidopsis TFs identified WRKY57 as a putative regulator of abscission. (A) Summary of TFs identified in the Y1H screen of the *IDA* promoter. TFs are represented by family. TF families with <4 members identified in the screen were grouped in the ‘Other’ category to facilitate visualization. See also Supplementary Table S4. (B) GO term enrichment analysis of the set of 211 TFs identified in the Y1H screen. GO terms associated with the 211 TFs were compared against the reference, in this case the entire list of TFs in *Arabidopsis thaliana*. All terms listed here are statistically enriched at *P*<0.01. Different colors represent related terms (blue, carbohydrates; green, ethylene; yellow, floral development; gray, lipids; orange, signaling; pink, other responses; white, DNA binding). (C) Histochemical staining of GUS activity in plants expressing GFP–GUS under the *WRKY57* promoter. Scale bar=0.4 mm. P5, P6, and P7 are floral positions 5, 6, and 7 along the main inflorescence stem. (D) Maximum intensity projections of confocal micrographs of the AZs of plants expressing *pWRKY57:WRKY57-GFP*. On the top panel, green nuclear signals from WRKY57–GFP are seen in the residuum cells. Scale bar=150 µm. Below, WRKY57–GFP accumulates in the secession cells of a petal. Scale bar=50 µm. Chlorophyll autofluorescence is shown in magenta in both images. (E) Inflorescences of T-DNA lines *wrky57*, *wrky48*, and the double mutant *wrky57 wrky48*. Scale bar=2 cm. (F) Quantitative phenotyping of floral organ abscission in the mutant lines listed in the key Markers (circles, squares, triangles) represent the mean, and whiskers the SEM. Data correspond to the number of floral organs fully attached to the flower at each position from five plants per genotype. One-way ANOVA and post-hoc Bonferroni test were used to compare the four genotypes at each position. Positions with a statistically significant difference in the mean compared with wild-type plants are highlighted with a white star (*P*<0.05). This assay was repeated twice with similar outcomes. (G) Irregular self-pollination in the *wrky57 wrky48* double mutant, highlighted by red arrows. Scale bar=1 cm.

Next, we aimed to identify and functionally characterize WRKYs that regulate *IDA* expression during floral organ abscission. We screened a collection of 2000 TFs of Arabidopsis in a Y1H assay to identify those that bound the *IDA* promoter. This screening detected interaction between the *IDA* promoter and 211 TFs, the majority of which belonged to the main TF types predicted with the *in silico* analysis (Fig. 3A; Supplementary Fig. S1). A GO enrichment analysis of the 211 TFs that bind the *IDA* promoter revealed enriched categories expected of abscission-related genes: floral whorl morphogenesis and carpel formation, sugar- and carbohydrate-mediated signaling, and regulation of ethylene responses, among others (Fig. 3B). Twelve WRKYs were detected in the Y1H screening, and most of them were induced in AZs as abscission progresses (Supplementary Fig. S7A). Out of these 12, WRKY57 and WRKY60 were selected as our primary candidates to regulate *IDA* because their own up-regulation preceded the induction of *IDA*, and *WRKY57* and *WRKY60* had been highlighted in the list of most significantly regulated genes of the AZ transcriptome (Cai and Lashbrook, 2008). Furthermore, WRKY57 is involved in balancing jasmonic acid and auxin signaling in leaves during senescence, is a negative regulator of biotic stress resistance, and its overexpression confers drought tolerance—processes previously linked to IDA signaling and abscission (Jiang *et al.*, 2012, 2014; Jiang and Yu, 2016; Serrano-Bueno *et al.*, 2022). Similarly, WRKY60 is involved in immunity, and abscisic acid (ABA) signaling in osmotic and salt stress (Xu *et al.*, 2006; Chen *et al.*, 2010; Liu *et al.*, 2012).

To verify the transcriptomic data *in planta*, transcriptional and translational fusion reporter lines were generated. Unexpectedly, the reporter lines of *WRKY60* indicated that both its promoter activity and its protein accumulation take place outside of the AZs, where WRKY60–GFP accumulates in the nucleus (Supplementary Figs S7B, C, S8A). A *wrky60* T-DNA insertion line was phenotyped at the flowering stage and no abscission defect was observed (Supplementary Fig. S7D). Based on this set of results, we discarded WRKY60 as a putative regulator of *IDA* in abscission. The promoter of *WRKY57* was active in AZs prior to abscission, and WRKY57–GFP accumulated in the nuclei of AZ cells, supporting a putative role for WRKY57 in regulating *IDA* and abscission (Fig. 3C, D; Supplementary Fig. S8B). Importantly, a single mutant *wrky57* abscised as wild-type plants (Fig. 3E). However, WRKYs are considered to be largely redundant with other members of their family (Du *et al.*, 2023). Functional redundancy is most common between proteins with the highest homology, and thus we generated a double knockout mutant between *WRKY57* and *WRKY48*, WRKY57’s closest homolog in BLAST searches against the Arabidopsis proteome (Altschul *et al.*, 1990; Jiang *et al.*, 2012). A delay in abscission was observed in *wrky57 wrky48* double mutants compared with the wild type, suggestive of a weak abscission defect (Fig. 3E, F). It must be noted that *wrky57 wrky48* mutants fail to fully fertilize some of their siliques (Fig. 3G), and so caution should be taken when concluding the cause of the abscission delay observed in this double mutant. Collectively, these findings portrayed WRKY57 as a potential regulator of *IDA* and abscission in redundancy with other WRKYs. This prompted us to functionally characterize WRKY57 activity in AZs.

### Overexpression of *WRKY57* or a *WRKY57* repressor respectively activates or represses floral organ abscission

**Fig. 4.**
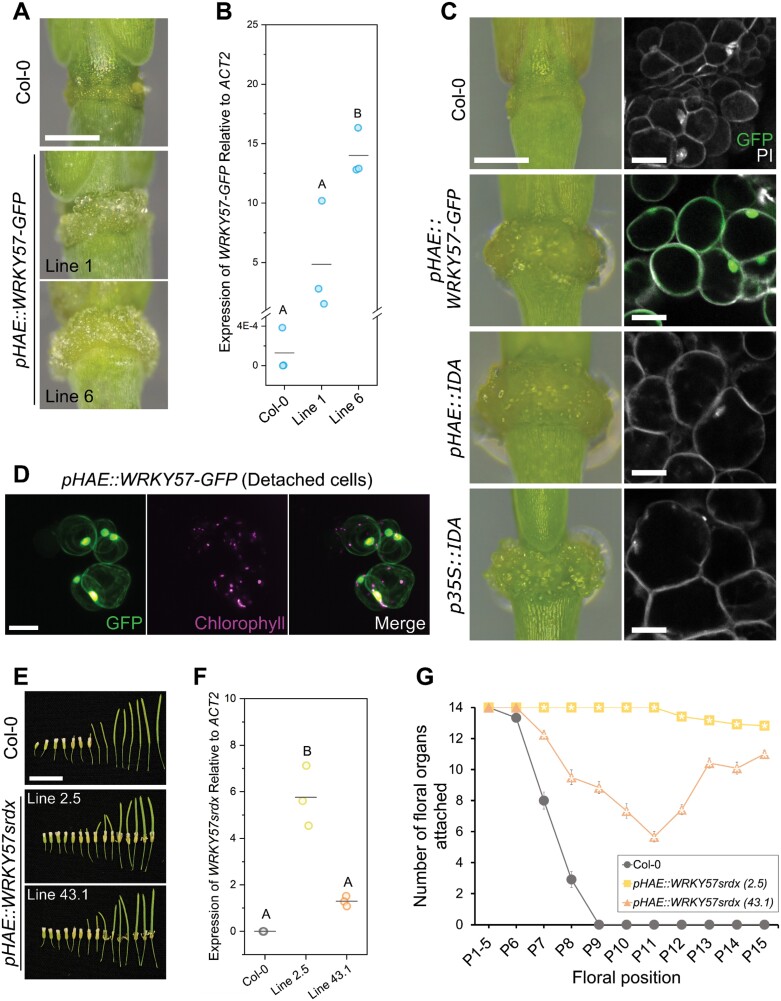
Modulation of floral organ abscission by local overexpression of *WRKY57*. (A) Floral receptacles of transgenic lines in the Col-0 background overexpressing WRKY57–GFP in floral position 12. Scale bar=0.4 mm. (B) Quantification of transgene expression levels in the transgenic lines shown in (A). Data points are one biological replica (averaged from two technical replicas). Three biological replicas were analyzed per line. Letters denote statistically different groups at *P*<0.05 according to one-way ANOVA and Bonferroni post-hoc tests. *ACT2* stands for *ACTIN2*. (C) Comparison of the AZ enlargement and cell expansion in lines locally overexpressing WRKY57, IDA, and the previously published constitutive *p35S::IDA*. See also Supplementary Fig. S9. Scale bars=0.4 mm for the panels on the left and 20 µm in the confocal images. Propidium iodide (PI) was used to stain the cell walls (and some nuclei). GFP is shown in green and PI in gray. (D) Example of cells that detach from the AZs of the *pHAE::WRKY57-GFP* lines while imaging. GFP fluorescence is shown in green, chlorophyll autofluorescence in magenta. Faint WRKY57–GFP signal can be observed in the cell periphery—most probably the cytoplasm—in samples that express the protein strongly. Scale bar=20 µm. (E) Plants locally overexpressing *WRKYsrdx* in AZs strongly retain their floral organs. Scale bar=1 cm. (F) Quantification of transgene expression levels in the transgenic lines shown in (E). The same description as in (B) applies. (G) Quantification of the floral organ abscission defect in the transgenic lines overexpressing *WRKY57srdx* in AZs. Markers (circle, square, triangle) represent the mean number of floral organs fully attached to the respective floral position. Whiskers are the SEM. White stars indicate statistically significant differences from wild-type Col-0 according to one-way ANOVA and post-hoc Bonferroni tests at *P*<0.05. Twelve plants per line were grown, and one flower per plant and floral position was analyzed.

TFs can positively or negatively regulate the transcription of genes. Typically, TFs bind TFBSs in the promoter region of genes, and recruit additional effectors to mediate activation or repression of transcription. WRKY57 was shown to induce or repress jasmonic acid-induced senescence in Arabidopsis leaves by competitively interacting with repressors from the JASMONATE ZIM-DOMAIN (JAZ) or AUX-IAA families, respectively (Jiang *et al.*, 2014). This functional duality indicates that the activity of WRKY57 is likely to be dependent on the tissue, developmental stage, and environmental cues, as is the abundance of interactors and hormones that influence WRKY57. To shed light on the role of WRKY57 during abscission, we locally overexpressed *WRKY57* using the *HAE* promoter—highly active during abscission in both residuum and secession cells (Jinn *et al.*, 2000; Lee *et al.*, 2018). Multiple transgenic lines overexpressing WRKY57–GFP in AZs displayed enlargement of the receptacle after abscission, correlating with the transgene level of expression (Fig. 4A, B; Supplementary Fig. S9A, B). The AZ enlargement was caused by excessive cell expansion, reminiscent of that observed in plants constitutively overexpressing *IDA* (*p35S::IDA*; Stenvik *et al.*, 2006), and those expressing *IDA* under the *HAE* promoter (*pHAE::IDA*; Fig. 4C; Supplementary Fig. S9C). The excessive cell expansion is probably due to overactive cell separation, as loose cells detach from the AZs of the WRKY57–GFP-overexpressing lines when mounting samples for microscopy (Fig. 4D).

Conversely, we overexpressed in AZs a chimeric WRKY57 fused to the SRDX repressor domain. The addition of the SRDX repressor domain to the C-terminus of a TF drives transcriptional repression of its target genes (Hiratsu *et al.*, 2003; Heyl *et al.*, 2008; Matsui and Ohme-Takagi, 2010; Mahfouz *et al.*, 2012; Cen *et al.*, 2016). Multiple independent lines expressing *WRKY57srdx* under the *HAE* promoter showed mild to very strong retention of floral organs in developing siliques, revealing a correlation between transgene expression level and phenotype severity (Fig. 4E–G). Opposite phenotypic outcomes on abscission when locally overexpressing WRKY57 or WRKY57srdx indicate that this TF acts as a positive regulator at the developmental stage in which AZ cells are competent to abscise. We observed induction and repression of the *IDA* promoter activity in luciferase transient transactivation assays in Arabidopsis leaf mesophyll protoplasts (Supplementary Fig. S10A). WRKY57 transactivation of the *IDA* promoter was shown to be partially dependent on the presence of the five WRKY TFBSs disrupted in the *WRKY*(–) version of this promoter (Supplementary Fig. S10B). These results suggest that WRKY57 positively regulates abscission by activating the *IDA* signaling pathway.

### Activation of abscission by WRKY57 requires IDA and redundant IDA-like peptides, as well as the receptors HAE and HSL2

**Fig. 5.**
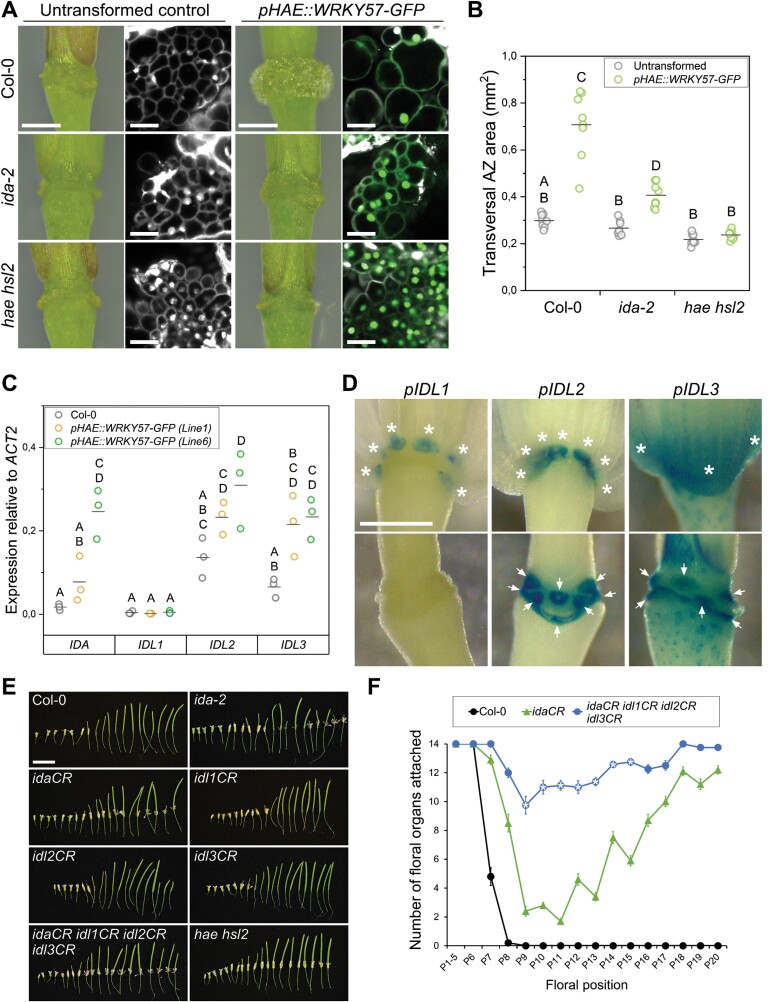
WRKY57 activates abscission via redundant *IDA* and *IDA*-*like* genes. (A) Comparison of the AZ enlargement and cell expansion in lines locally overexpressing WRKY57–GFP in wild-type, *ida-2*, and *hae hsl2* mutant backgrounds. See also Supplementary Fig. S11. Scale bars=0.4 mm for the panels on the left and 20 µm in the confocal images. GFP is shown in green and PI in gray. (B) Quantification of the transversal AZ area in the genotypes displayed below the graph, either untransformed, or expressing the *pHAE::WRKY57-GFP* transgene. Data correspond to the measurement of the position 12 AZ area in the main inflorescence stem for each plant. Eight plants per genotype were analyzed. Letters show statistically significant differences between groups according to one-way ANOVA and Bonferroni post-hoc analyses (*P*<0.05). (C) Estimation of gene expression of *IDA*, *IDL1*, *IDL2*, and *IDL3* in the genotypes listed in the key. Data correspond to the average of two technical replicates per biological replica. Three biological replicas were assayed per genotype. Letters show statistically significant differences between groups according to two-way ANOVA and Bonferroni post-hoc analyses (*P*<0.05). (D) Histochemical detection of GUS activity in plants expressing GFP–GUS under the control of the promoters listed over the panels. White stars highlight the floral organs still attached in the upper panels. White arrows point at the residuum cells with clear GUS activity in the receptacle. Scale bar=0.4 mm. (E) Floral organ abscission in the genotypes listed in the panels. Scale bar=1 cm. (F) Quantification of retained floral organs per floral position along the main inflorescence stem for the genotypes listed in the key. Markers (circles and triangles) represent the mean; whiskers are the SEM. White stars denote the floral positions of the quadruple *idaCR idl1CR idl2CR idl3CR* that were significantly different from *idaCR* according to one-way ANOVA and Bonferroni post-hoc tests (*P*<0.05). See Supplementary Fig. S12 for the expanded quantification of all genotypes included in (E).

Next, we explored the epistasis between the *pHAE::WRKY57-GFP* transgene, *IDA*, and *HAE/HSL2*. We crossed the *ida-2* and *hae hsl2* mutants to the single copy line *pHAE::WRKY57-GFP* (line 6), and obtained double homozygous *ida-2 pHAE::WRKY57-GFP* and triple homozygous *hae hsl2 pHAE::WRKY57-GFP* plants. Microscopic examination of the receptacle and AZ cells confirmed that the effect of the local overexpression of *WRKY57* in AZs depends on *IDA* and the receptor genes *HAE* and *HSL2* (Fig. 5A). Plants lacking functional receptors HAE/HSL2 completely suppressed the AZ enlargement and cell expansion phenotypes; however, a weak but statistically significant enlargement was observed in the *ida-2 pHAE::WRKY57-GFP* double mutants (Fig. 5B). We sought to independently confirm this result by transforming *ida-1* and *ida-2* alleles of *IDA* with the *pHAE::WRKY57-GFP* construct. Multiple independent lines displayed varying degrees of AZ enlargement, and the enlargement correlated with a complementation of the abscission defect of *ida*, suggesting that induction of abscission by WRKY57 is only partially dependent on *IDA* while it is fully dependent on *HAE/HSL2* (Supplementary Fig. S11A). We reasoned that WRKY57 could also activate HAE/HSL2 via *IDL1*, *IDL2*, or *IDL3*—close homologs of *IDA*. These three *IDL* genes contain several WRKY TFBSs in their promoter sequences, were reported to be expressed in floral receptacles, and were expected to play a redundant role with *IDA* in abscission (Supplementary Fig. S11B; Vie *et al.*, 2015). Transcriptional up-regulation of *IDA*, *IDL2*, and *IDL3* was detected by RT–qPCR analysis from dissected floral receptacles in *pHAE::WRKY57-GFP* lines, and the capacity of WRKY57 to transactivate *IDL* genes was confirmed in transient luciferase assays in *N. benthamiana* leaves with the *IDL2* promoter (Fig. 5C; Supplementary Fig. S11C). These findings support a model where WRKY57 works as a positive regulator of abscission by orchestrating the coordinated expression of several redundant IDL peptides to activate the receptors HAE/HSL2.

The abscission-inducing activity of *IDL1*, *IDL2*, and *IDL3* was demonstrated in constitutive overexpression lines (Stenvik *et al.*, 2008). IDL2 and IDL3 were shown to bind with high affinity to the complex formed by HAE and SERK extracellular domains, as well as the extracellular domain of HSL1—the closest homolog to HAE/HSL2 in Arabidopsis (Roman *et al.*, 2022). Genetic evidence involving *IDL1*, *IDL2*, and *IDL3* in floral organ abscission has nevertheless remained elusive. Promoter reporter lines for *IDL1*, *IDL2*, and *IDL3* expressing GUS–GFP were generated and their previously reported expression patterns during abscission confirmed (Stenvik *et al.*, 2008; Vie *et al.*, 2015). *IDL1* promoter activity was detected in the secession cells only, while in the case of *IDL2* and *IDL3*, promoter activities were detected in both residuum and secession cell layers (Fig. 5D). Gene-edited knockout lines for *IDL2* and *IDL3* were generated in the Col-0 background with the CRISPR/Cas9 [clustered regularly interspaced palindromic repeats (CRISPR)/CRISPR-associated protein 9] technology, and higher order mutants were obtained by genetic crosses with previously published *idaCR* and *idl1CR* (Shi *et al.*, 2018; Alling and Galindo-Trigo, 2023). The *idaCR* line was used despite the availability of the *ida-2* allele, also in Col-0, due to T-DNA-induced genomic rearrangements in the *ida-2* line that impede obtaining double mutants between *ida* and *idl1* alleles (Alling and Galindo-Trigo, 2023). Developing siliques in the quadruple mutant *idaCR idl1CR idl2CR idl3CR* retained floral organs more strongly than the single *ida* mutant, confirming the genetic redundancy between these IDL peptides as positive regulators of abscission (Fig. 5E, F; Supplementary Fig. S12). Based on the cumulative evidence provided here, we propose a model in which WRKY57 modulates the transcription of *IDA* and *IDL* genes to fine-tune the timing of floral organ abscission.

## Discussion

The multiplicity of cues affecting abscission and the expansion of TF families in flowering plants make characterizing transcriptional effectors in this process a demanding task. In this study, we show that the transcriptional regulation of *IDA*, one of the main triggers of floral organ abscission, is subject to the control of WRKY TFs (Fig. 1). Using molecular genetics and physiological assays, we showed that the IDA signaling pathway can be activated upon exposure to a bacterial immune elicitor in AZs, and that this activation depends on WRKY TFBSs in the *IDA* promoter (Fig. 2). Screening a large collection of TFs in a heterologous system, we identified a list of potential candidates to directly regulate *IDA* expression, and genetically characterized WRKY57 as such (Figs 3, 4, 5A, B). Finally, by locally overexpressing WRKY57 in AZs, we found evidence of coordinated transcriptional regulation of *IDA*, *IDL2*, and *IDL3* (Fig. 5C). The generation of higher order *ida/idl* mutants confirmed the long-standing hypothesis of functional redundancy among these genes (Fig. 5E, F).

ERFs are known to relay the transcriptional signaling induced by ethylene downstream of the master transcriptional regulator ETHYLENE INSENSITIVE3 (EIN3; Chao *et al.*, 1997; Chang *et al.*, 2013). Indeed, the result of deleting 33 bp in the *IDA* promoter containing an ERF TFBS yielded the same expression pattern as observed for the wild-type *IDA* promoter in an ethylene-insensitive background (Butenko *et al.*, 2006). We speculate that this regulation could be exerted by ERFs such as *AT5G25190*, found to bind the *IDA* promoter in the Y1H screen and selected as one of the most likely genes to regulate floral organ abscission by Cai and Lashbrook (2008) based on its transcriptional profile. Detailed analysis of all ERF TFBSs in the *IDA* promoter and functional characterization of promising ERFs during abscission should shed light on this matter. Interestingly, the master regulator of ethylene, EIN3, was identified in the Y1H screening as a TF that binds the *IDA* promoter (Supplementary Table S4). EIN3 up-regulates the transcription of its direct targets, providing further support for the model in which *IDA* is activated by ethylene signaling during floral organ abscission (Chang *et al.*, 2013; Meir *et al.*, 2019). Although *IDA* was not detected in the genome-wide screen for EIN3-mediated, ethylene-induced transcriptionally regulated genes in Arabidopsis, developmental or tissue-specific effects could explain its absence, as these experiments were conducted in 3-day-old etiolated seedlings (Chang *et al.*, 2013). Dissecting the importance and the mechanism behind the putative two-tiered regulation of *IDA* by EIN3 and ERFs will be the topic of future investigations.

We have shown that residuum cells of recently abscised floral receptacles (positions 6 or 7) respond to flg22 treatments by up-regulating *IDA* (Fig. 2A, B). While the fluorescent reporter data suggest that residuum cells are particularly responsive to flg22, this responsiveness pattern may not reflect responsivity *per se*, but rather their capacity to take up the treatment. Residuum cells synthesize a cuticle after abscission takes place, although in the floral positions analyzed here this process may not have concluded, presenting a less hydrophobic exterior and increased permeability. Regardless of the spatial specificity of the response, the responsivity of the *IDA* promoter in residuum cells to biotic stress after organs abscise has interesting implications. Firstly, it reinforces the notion that IDA signaling is activated in response to flg22 in cells destined for separation. Secondly, reactivation of IDA signaling in the cells that control cell separation will probably lead to the activation of a similar set of responses triggered during abscission, as the expression of receptors HAE/HSL2 and other downstream regulators remains active in residuum cells after separation (Cho *et al.*, 2008). Flg22 elicitation of *IDA* expression could induce a protective shedding mechanism that is useful for the plant when still-attached floral organs are infected by bacterial pathogens, similar to pathogen-induced cauline leaf shedding (Patharkar *et al.*, 2017). Alternatively, activating IDA signaling could help residuum cells protect themselves against pathogens by reinforcing the flg22-mediated signaling. When seedlings are exposed to exogenous IDA peptide, defense response marker genes that are typically induced by flg22 such as *FLG22-INDUCED RECEPTOR-LIKE KINASE1* (*FRK1*; Asai *et al.*, 2002) are transcriptionally up-regulated. When seedlings are exposed to both IDA and flg22, the expression of these marker genes is up-regulated even further (Lalun *et al.*, 2023). This suggests a role for IDA in enhancing the flg22-mediated transcriptional response, something potentially beneficial for residuum cells to protect them as cell separation occurs. There is, however, conflicting evidence that indicates a negative role during leaf bacterial colonization for the IDA receptors HAE/HSL2 and the related peptide IDL6 (Wang *et al.*, 2017). Understanding the physiological consequence of the molecular signatures triggered by IDA treatments that overlap with immune responses awaits further analyses. AZ-centric physiological studies will provide exciting new insights into the relationship between abscission and immunity.

Out of the 12 WRKYs found in the Y1H screen on the *IDA* promoter, there are several good candidates to regulate *IDA* during biotic stress. In ChIP-seq and MS analyses of nuclear proteins, Birkenbihl *et al.* (2018) reported the *IDA* promoter-binding TFs WRKY8 and WRKY11 to be both transcriptionally up-regulated and to increase in protein abundance upon flg22 treatments. WRKY8 was found to execute most of the transcriptional responses in chitin-induced, PBS1-LIKE 19 (PBL19)-mediated immunity. Upon chitin treatments—also known to induce *IDA* expression, PBL19 accumulates in the nucleus, interacts and phosphorylates WRKY8, which in turn transcriptionally up-regulates genes. Transgenic plants expressing a version of PBL19 that constitutively accumulates in the nucleus show a severe autoimmunity phenotype, which is dependent on WRKY8. An RNA-seq analysis of the autoimmune PBL19-expressing plants revealed that *HAE*, *IDL6*, and *IDL7* were up-regulated along with immunity marker genes such as *FRK1*—data for the *IDA* gene were unfortunately not available in the dataset (Li *et al.*, 2022). This suggests that upon chitin perception, WRKY8 induces expression of components of the IDA signaling pathway, and quite possibly also *IDA*. Since WRKY8 increases in abundance upon flg22 detection, it is likely that a similar set of genes are up-regulated by WRKY8 in the context of flg22 perception, making WRKY8 a prime candidate to mediate the flg22-induced IDA expression. WRKY11, on the other hand, acts as a negative regulator of gene expression and inhibits transcription of genes typically induced in the context of immunity redundantly with WRKY7, WRKY15, WRKY17, WRKY21, and WRKY39. Quintuple mutants *wrky7-11-17-21-39* show severe developmental defects and display constitutive up-regulation of defense-related genes. An exploration of the RNA-seq dataset comparing *wrky7-11-17-21-39* with the wild type confirmed that the IDA signaling pathway is also differentially expressed, with *IDA*, *HAE*, *IDL6*, and *IDL7* being up-regulated in the quintuple *wrky* mutant (Du *et al.*, 2023). This set of WRKYs probably constitute a negative feedback loop to the defense-induced transcriptional responses in the plant, including components of the IDA signaling pathway.

We have genetically characterized WRKY57 as a potential positive regulator of abscission, exerting its transcriptional control on *IDA*, *IDL2*, and *IDL3* (Figs 4, 5). In rosette leaves, WRKY57 can be co-opted by repressors from the jasmonic acid and auxin signaling pathways to respectively induce or repress jasmonic acid-induced senescence (Jiang *et al.*, 2014). Both hormones have important roles in the abscission and senescence of floral organs. While auxin inhibits abscission (Basu *et al.*, 2013), jasmonic acid signaling regulates floral organ senescence and the timing of abscission (Kubigsteltig *et al.*, 1999; Serrano-Bueno *et al.*, 2022). Thus, we speculate that WRKY57 could help orchestrate the coordinated transcriptional regulation of the *IDA* gene from auxin and jasmonic acid signaling pathways to fine-tune abscission timing.

The regulatory axis described in this study comprising WRKY–IDA signaling–abscission is not exclusive to floral organ abscission in Arabidopsis. Tomato pedicels have been recently shown to utilize SlWRKY17 (closely related to *A. thaliana* WRKY6, WRKY31, and WRKY42—members of a different WRKY subfamily from WRKY57/48) to mediate low light-induced pedicel abscission by regulating tomato *IDL6* gene expression (Li *et al.*, 2021). This demonstrates that the relationship between WRKY TFs and abscission is conserved across plant species and in diverse plant organs.

Introducing beneficial traits into crops by targeted genome editing is easier than ever; however, gene functions are normally multifaceted and involved in multiple processes. Our study exemplifies how promoter element modification can be exploited to allow the developmentally encoded expression profile of a gene to proceed, while impairing its responsiveness to stress (Figs 1, 2). Promoter-targeted genome editing approaches therefore show promise in allowing the introduction of beneficial traits such as controlled abscission into crops, while minimizing developmental penalties to yield (Liu *et al.*, 2021; Tang and Zhang, 2023; Zhou *et al.*, 2023).

## Supplementary data

The following supplementary data are available at *JXB* online.

Table S1. Primers used in this study.

Table S2. Transcription factor-binding site prediction for the *IDA* promoter.

Table S3. *pIDA* promoter sequences.

Table S4. Transcription factors identified to bind the *IDA* promoter in the yeast one-hybrid screening.

Fig. S1. Transcription factor-binding site analysis of the *IDA* promoter sequence in Arabidopsis.

Fig. S2. Additional information to Fig. 1B.

Fig. S3. Histochemical detection of GUS activity during lateral root emergence.

Fig. S4. The *ERF*(–) version of the *IDA* promoter cannot rescue the abscission defect of the *ida-1* mutant.

Fig. S5. Deletion of individual WRKY-binding sites in the *IDA* promoter does not impair its activity during abscission.

Fig. S6. Elicitation of immune responses in cauline leaves with flg22 does not induce *IDA* in the cauline AZ.

Fig. S7. Additional information to Fig. 3.

Fig. S8. Nuclear localization of the fusion proteins WRKY60–GFP and WRKY57–GFP.

Fig. S9. Quantification of the receptacle enlargement phenotype shown in Fig. 4.

Fig. S10. Transient transactivation of the *IDA* promoter by WRKY57 in Arabidopsis mesophyll protoplasts.

Fig. S11. WRKY57 activates the abscission pathway in *ida* mutants via IDL peptides.

Fig. S12. Quantification of floral organ abscission at positions 8, 10, 12, 16, and 20.

erae014_suppl_Supplementary_Tables_S1

erae014_suppl_Supplementary_Tables_S2

erae014_suppl_Supplementary_Tables_S3

erae014_suppl_Supplementary_Tables_S4

erae014_suppl_Supplementary_Figures_S1-S12

## Data Availability

All data presented can be found in the manuscript and its supplementary data. Requests for further details can be directed to the corresponding authors.

## Abbreviations

AZ: abscission zone
ERF: ethylene response factor
HAE: HAESA
IDA: INFLORESCENCE DEFICIENT IN ABSCISSION
IDL: IDA-like
MAMP: microbe-associated molecular pattern
TF: transcription factor
TFBS: TF-binding site
Y1H: yeast one-hybrid
